# Toxicity evaluation of Wanzhou watershed of Yangtze Three Gorges Reservoir in the flood season in *Caenorhabditis elegans*

**DOI:** 10.1038/s41598-018-25048-w

**Published:** 2018-04-30

**Authors:** Guosheng Xiao, Li Zhao, Qian Huang, Junnian Yang, Huihui Du, Dongqin Guo, Mingxing Xia, Guangman Li, Zongxiang Chen, Dayong Wang

**Affiliations:** 1 College of Biology and Food Engineering, Chongqing Three Gorges University, Wanzhou, 404100 China; 2 Medical School, Southeast University, Nanjing, 210009 China; 3 Wanzhou Entry-Exit Inspection and Quarantine Bureau, Wanzhou, 404100 China

## Abstract

Three Gorges Reservoir (TGR) in the upper stream of Yangtze River in China is a reservoir with the largest and the longest yearly water-level drop. Considering the fact that most of safety assessments of water samples collected from TGR region were based on chemical analysis, we here employed *Caenorhabditis elegans* to perform *in vivo* safety assessment of original surface water samples collected from TGR region in the flood season in Wanzhou, Chongqing. Among the examined five original surface water samples, only exposure to original surface water sample collected from backwater area could induce the significant intestinal ROS production, enhance the intestinal permeability, and decrease the locomotion behavior. Additionally, exposure to original surface water sample collected from backwater area altered the expressions of *sod-2*, *sod-5*, *clk-1*, and *mev-1*. Moreover, mutation of *sod-2* or *sod-5* was susceptible to the potential toxicity of original surface water sample collected from backwater area on nematodes. Together, our results imply that exposure to surface water sample from the backwater area may at least cause the adverse effects on intestinal function and locomotion behavior in nematodes.

## Introduction

Construction of Three Gorges Dam created a novel ecosystem in the upper stream of the Yangtze River in China. Three Gorges Reservoir (TGR) is the world’s largest reservoir with the largest and the longest yearly water-level drop. This reservoir is from Chongqing (west) to Yichang of Hubei province (east), and the distance is approximately 662.9 km^1^. In the winter, water level of the TGR is 175 m, whereas water level of the TGR is only 145 m in the summer for the aim of flood control^2^. This water fluctuation every year produces an area of 400 km^2^ along the upper stream of Yangtze River called water-level fluctuating zone (WLFZ). Meanwhile, it has been gradually recognized that the water fluctuation in TGR region may potentially affect the environmental and the ecological processes, i.e., sediment deposition, ecological sensitivity, soil erosion, and nutrient release^3,4^. More importantly, the potential pollution from both organic and inorganic pollutants may also be formed in the TGR region^5–7^.

With the rapid development of industrialization and urbanization, the TGR region along Yangtze River would receive a large amount of industrial and residential wastewater and release the large sources of water pollutants into the Yangtze River^8^. In freshwater ecosystems in TGR region, dissolved heavy metals, organic pollutants, surface-suspended particulate matter, and microplastic pollutant have been detected^5,7,9–11^. Bacterioplankton assemblage analysis in the TGR region further revealed the ecological and the spatial-temporal variations in bacterioplankton community composition^12,13^. Several reports have implied that the water fluctuation in TGR region may cause the disruptions on algal biomass, species composition, diversity, and richness^14–16^.

Nematodes are the most abundant metazoan in the soil environment. A free-living nematode, *Caenorhabditis elegans*, is a simple multicellular eukaryote^17^. Meanwhile, its sensitivity to different toxicants or stresses makes it suitable to offer a model for asking *in vivo* toxicological questions^18–27^. Besides the endpoint of lethality, some useful sublethal endpoints, such as lifespan, development, reproduction, locomotion behavior, and oxidative stress, have been employed to assess the toxicity from different environmental toxicants^28–32^. More importantly, several reports have demonstrated that *C*. *elegans* can be used for ecological risk assessment in water or in sediments^33–42^.

So far, most of the data on safety assessment of water samples from TGR region has been analyzed based on chemical analysis^5,11,43^. In contrast, the exposomics is urgently used to effectively assess the safety issue in the environment in the TGR region^44^. In China, Chongqing is a city with heavy industry in the central urban area. Wanzhou is further one of the main urban areas in Chongqing in the TGR region. In the present study, we employed the *C*. *elegans* to perform the *in vivo* ecotoxicological assessment of Wanzhou watershed in the flood season in the TGR region. Our study provides an important highlight for our understanding the ecotoxicological risk of watershed in the flood season in the TGR region.

## Results

### Effects of different surface water samples in the TGR region on survival and body length

**Figure 1:**
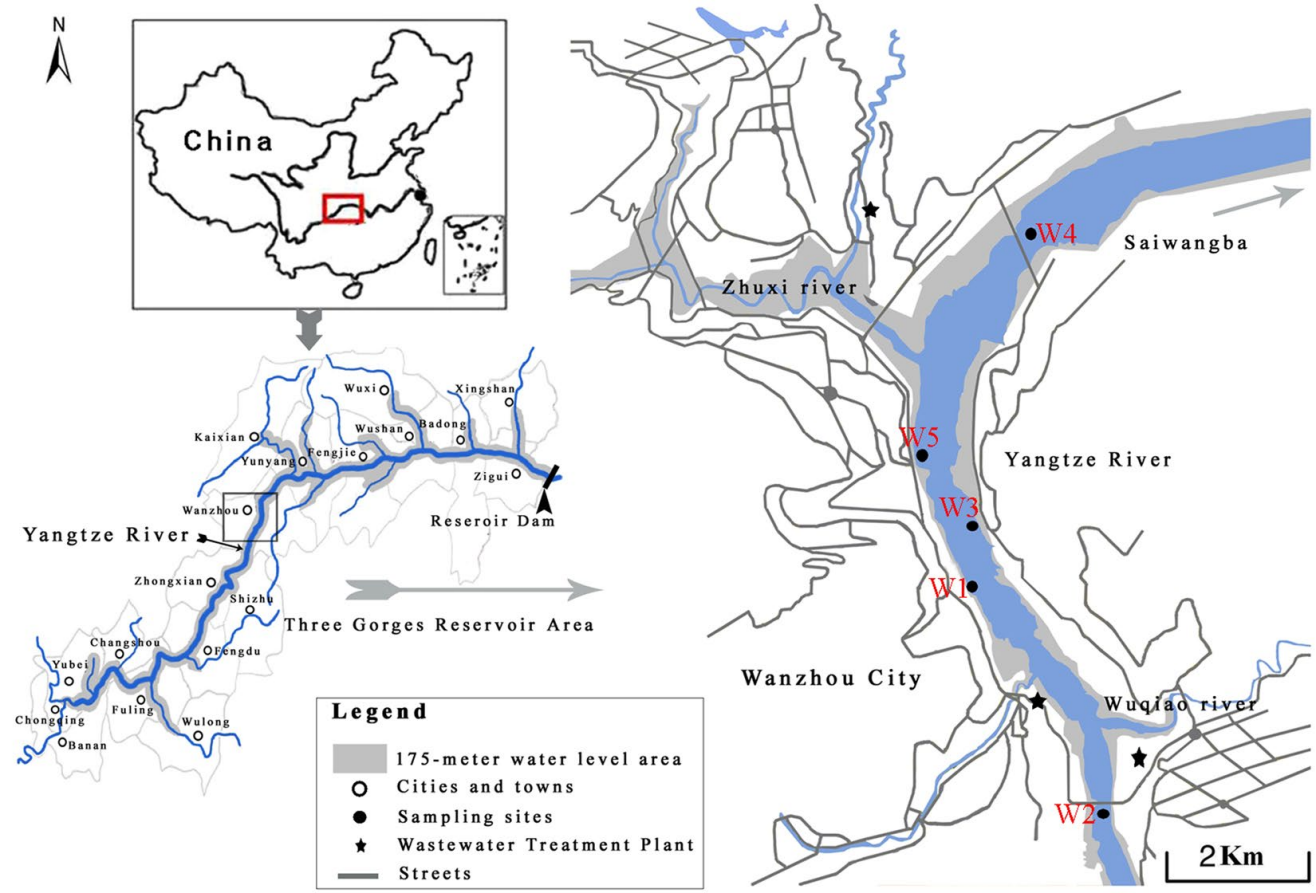
Sampling sites in the Three Gorges Reservoir.

**Figure 2:**
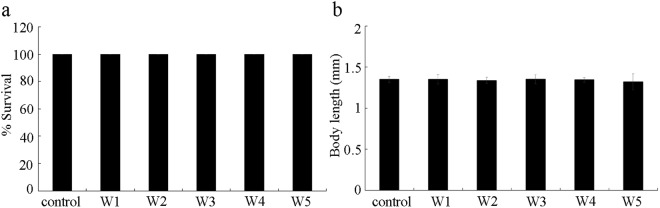
Effects of different surface water samples in the TGR region on survival and development of wild-type nematodes. (**a**) Effects of different surface water samples on survival. (**b**) Effects of different surface water samples on development. Development was assessed by the endpoint of body length. Exposures were performed from L4-larvae for 24-h. Bars represent means ± SD.

In this study, the surface water samples were from 5 sampling sites (W1, shore water; W2, upstream; W3, shore water; W4, downstream; and W5, backwater area) in Wanzhou, Chongqing (Fig. 1). We first investigated the effects of different surface water samples from TGR region on survival and development of wild-type nematodes. Exposures were performed from L4-larvae for 24-h (acute exposure) in liquid solutions in the presence of food (OP50). After exposure, we found that all the examined surface water samples from TGR region did not affect the survival of wild-type nematodes (Fig. 2a). Similarly, all the examined surface water samples from TGR region could not significantly influence the body length of wild-type nematodes (Fig. 2b). Therefore, all the examined surface water samples from TGR region may be not able to affect the survival and body length in wild-type nematodes.

### Effect of different surface water samples in the TGR region on induction of oxidative stress

**Figure 3:**
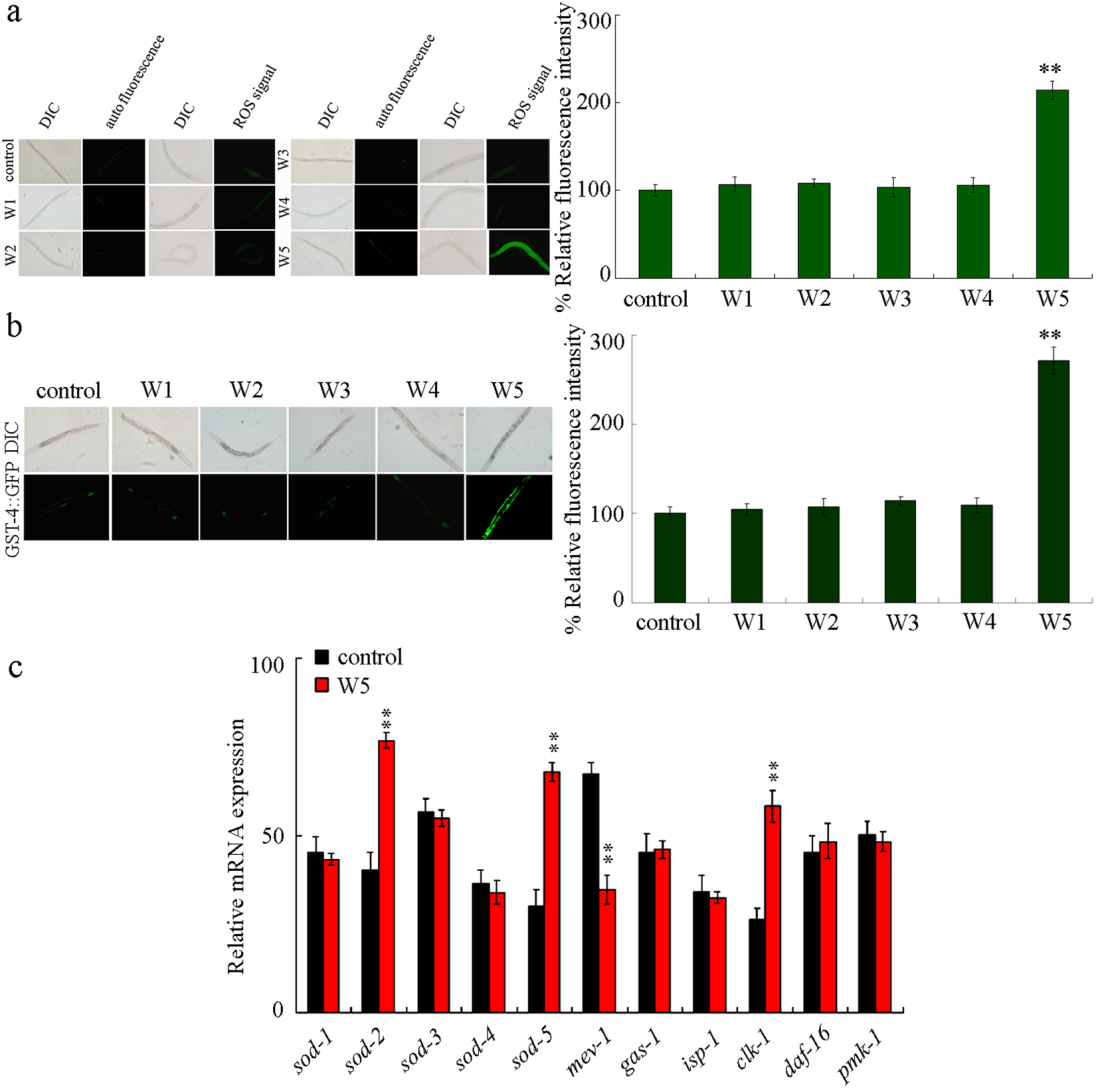
Effect of different surface water samples in the TGR region on induction of oxidative stress in wild-type nematodes. (**a**) Effect of different surface water samples on induction of intestinal ROS production. The left shows the pictures of intestinal ROS production, and the right indicates the comparison of relative fluorescence intensity for signals labeling ROS production in intestine. (**b**) Effect of different surface water samples on GST-4::GFP expression. Thirty animals were analyzed per treatment. (**c**) Comparison of expression patterns for genes required for oxidative stress. Exposures were performed from L4-larvae for 24-h. Bars represent means ± SD. ^**^P < 0.01 *vs* control.

Oxidative stress is normally considered as a common mechanism for the toxicity formation from environmental toxicants or stresses in nematodes^18,45–47^. No obvious production of intestinal reactive oxygen species (ROS) was detected in wild-type nematodes after exposure to surface water sample of W1, W2, W3, or W4 (Fig. 3a). In contrast, we found the severe intestinal ROS production after exposure to the surface water sample W5 (Fig. 3a).

GST-4, a putative glutathione-requiring prostaglandin D synthase, can be increased by exposure to paraquat, a ROS generator, and act as an oxidative stress-response protein in *C*. *elegans*^48^. We further used the transgenic strain of GST-4::GFP to determine the role of examined surface water samples in inducing ROS production. We did not found the obvious alteration in GST-4::GFP expression after exposure to the surface water sample of W1, W2, W3, or W4 (Fig. 3b). Different from this, we observed the expressional increase in the GST-4::GFP after exposure to surface water sample W5 (Fig. 3b).

In nematodes, genes encoding superoxide dismutases (SODs) (*sod-1-5*), subunits of mitochondrial complex or components in electron transfer chain (*isp-1*, *mev-1*, *clk-1*, and *gas-1*), insulin signaling (*daf-16*), and p38 mitogen-activated protein kinase (p38 MAPK) signaling (*pmk-1*) are involved in the regulation of oxidative stress^49–56^. After exposure to the surface water sample of W5, no alteration in transcriptional expressions of *isp-1*, *gas-1*, *sod-1*, *sod-3*, *sod-4*, *daf-16*, and *pmk-1* was detected (Fig. 3c). In contrast, exposure to the surface water sample of W5 increased expressions of *sod-2*, *sod-5*, and *clk-1*, and decreased *mev-1* expression (Fig. 3c). *sod-2* encodes a manganese SOD, and *sod-5* encodes a copper/zinc SOD.

The insulin signaling pathway can normally regulate the biological processes, such as longevity, through limiting the DAF-16 nuclear localization^57^. Nevertheless, with the aid of transgenic strain of *zIs356*, we found that exposure to all the examined surface water samples could not induce the obvious increase in DAF-16:GFP expression in the nuclei (data not shown).

### Effect of different surface water samples in the TGR region on intestinal permeability

**Figure 4:**
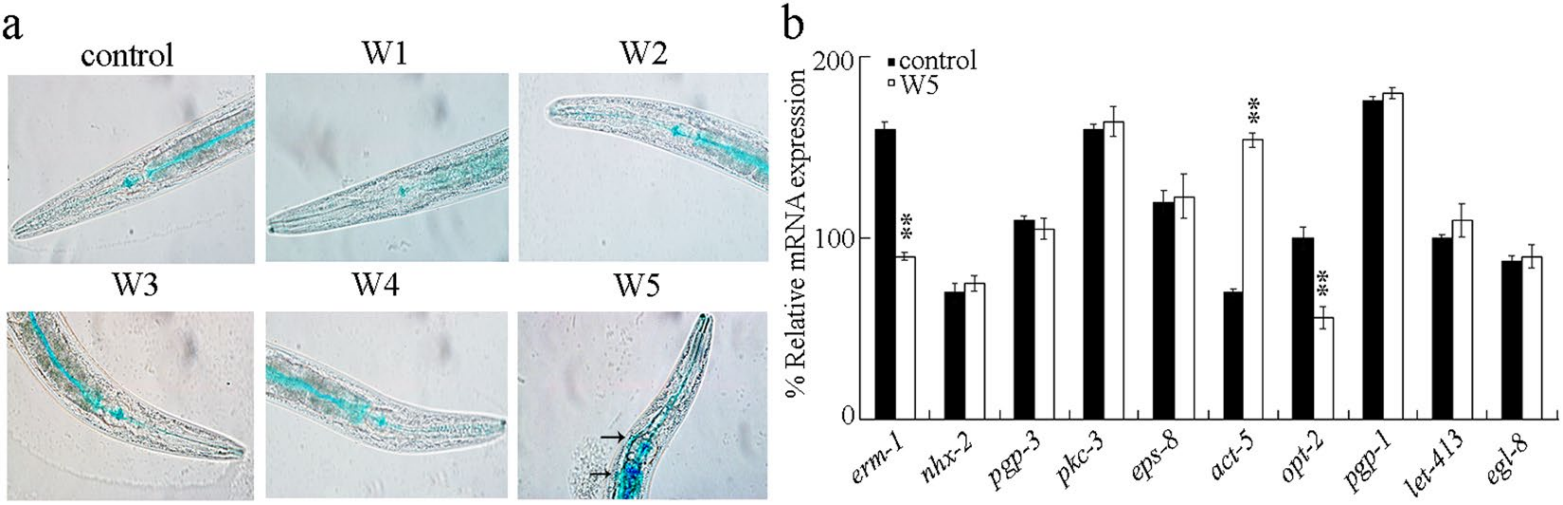
Effect of different surface water samples in the TGR region on intestinal permeability in wild-type nematodes. (**a**) Analysis on the dye distribution and leakage from the intestinal lumen into the body cavity. Arrowheads indicate the dye leakage from the intestinal lumen into the body cavity. (**b**) Effect of exposure to the sample of W5 on expressions of genes required for the control of intestinal development. Exposures were performed from L4-larvae for 24-h. Bars represent means ± SD. ^**^P < 0.01 *vs* control.

We further investigated the possible effect of surface water samples in the TGR region on the function of intestine based on the assay of the state of intestinal permeability. Using a non-absorbable blue food dye^58^, we measured the intestinal barrier function after exposure to the examined surface water samples. In control nematodes, the dye was mainly distributed within the intestinal lumen (Fig. 4a). Similarly, after exposure to the surface water sample of W1, W2, W3, or W4, the dye was also mainly accumulated within the intestinal lumen (Fig. 4). Different from this, exposure to the surface water sample of W5 caused the obvious dye leakage from the intestinal lumen into the body cavity (Fig. 4a), suggesting the potential decline in intestinal integrity in wild-type nematodes exposed to the surface water sample of W5.

Moreover, exposure to the surface water sample of W1, W2, W3, or W4 did not significantly affect the transcriptional expressions of *egl-8*, *eps-8*, *act-5*, *opt-2*, *nhx-2*, *pgp-1*, *let-413*, *pkc-3*, *erm-1*, and *pgp-3*, which are required for the control of intestinal development^25^ (data not shown). Meanwhile, exposure to the surface water sample of W5 also did not significantly affect the transcriptional expressions of *egl-8*, *eps-8*, *nhx-2*, *pgp-1*, *let-413*, *pkc-3*, and *pgp-3* (Fig. 4b). In contrast, exposure to the surface water sample of W5 significantly decreased the transcriptional expressions of *erm-1* and *opt-2*, and increased the transcriptional expression of *act-5* (Fig. 4b). In nematodes, ACT-5, ERM-1, EPS-8, PKC-3, OPT-2, NHX-2, PGP-1, and PGP-3 are required for the control of development of intestinal apical domain, LET-413 is required for the control of development of intestinal basolateral domain, and EGL-8 is required for the control of development of intestinal apical junctions. These data implies the possible deficit in development of intestinal apical domain, which further provides an important molecular basis for the intestinal dysfunction in nematodes exposed to the sample of W5.

### Effect of different surface water samples in the TGR region on reproduction and locomotion behaviour

**Figure 5:**
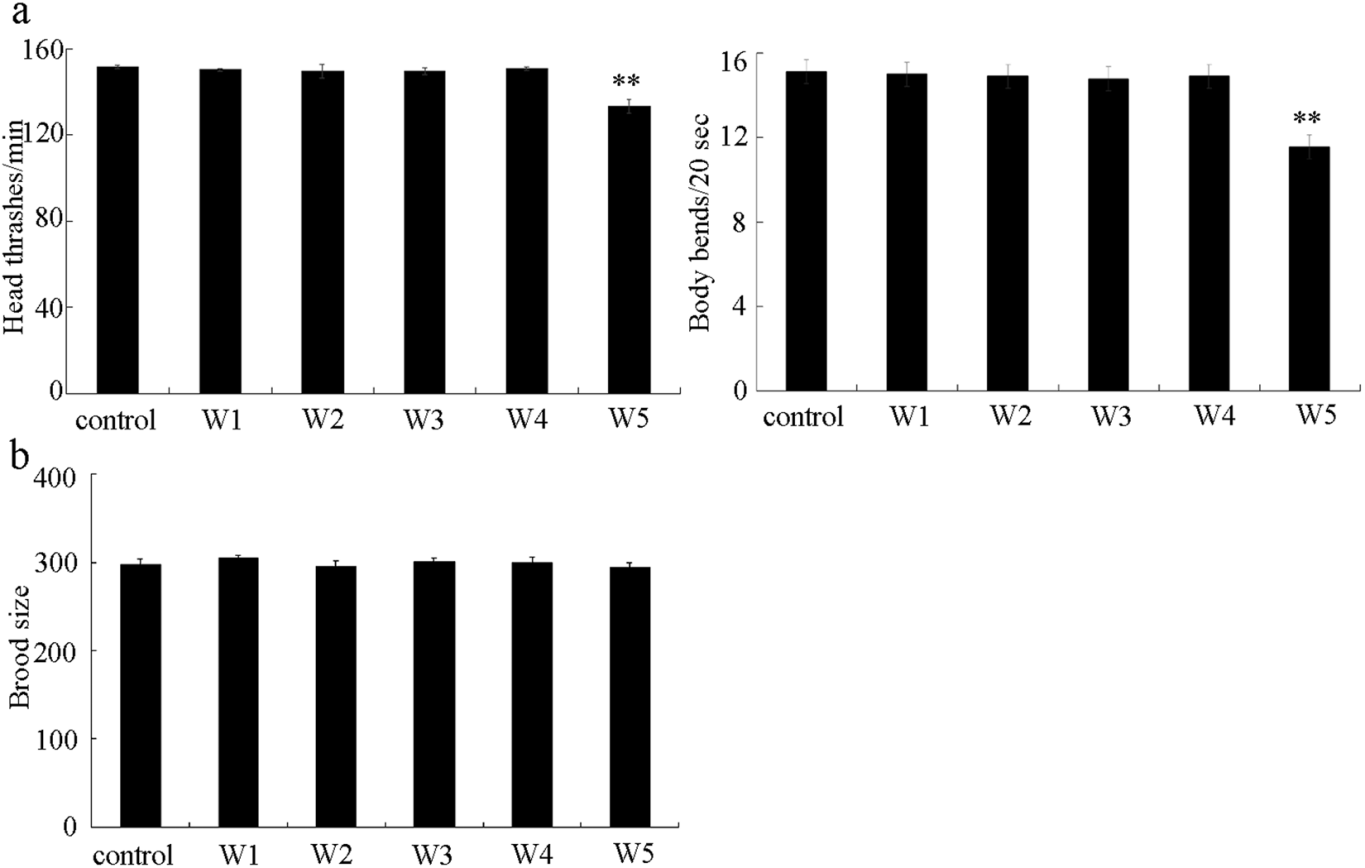
Effects of different surface water samples in the TGR region on locomotion behavior and reproduction in wild-type nematodes. (**a**) Effect of different surface water samples on locomotion behavior. Locomotion behavior was assessed by the endpoints of head thrash and body bend. (**b**) Effect of different surface water samples on reproduction. Reproduction was assessed by the endpoint of brood size. Exposures were performed from L4-larvae for 24-h. Bars represent means ± SD. ^**^P < 0.01 *vs* control.

In nematodes, neurons and reproductive organs are important secondary targeted organs, once the environmental toxicants can be translocated through the intestinal barrier^59^. After exposure, surface water sample of W1, W2, W3, or W4 could not significantly alter both the head thrash and the body bend (Fig. 5a). In contrast, exposure to surface water sample of W5 significantly decreased both the head thrash and the body bend (Fig. 5a). Therefore, among the examined five surface water samples in the TGR region, exposure to W5 may cause the alteration in function of motor neurons.

Different from the effect of surface water samples in the TGR region on locomotion behavior, we found that all the examined surface water samples (W1, W2, W3, W4, and W5) could not significantly affect the brood size of wild-type nematodes (Fig. 5b).

### Effect of different surface water samples in the TGR region on lifespan

**Figure 6:**
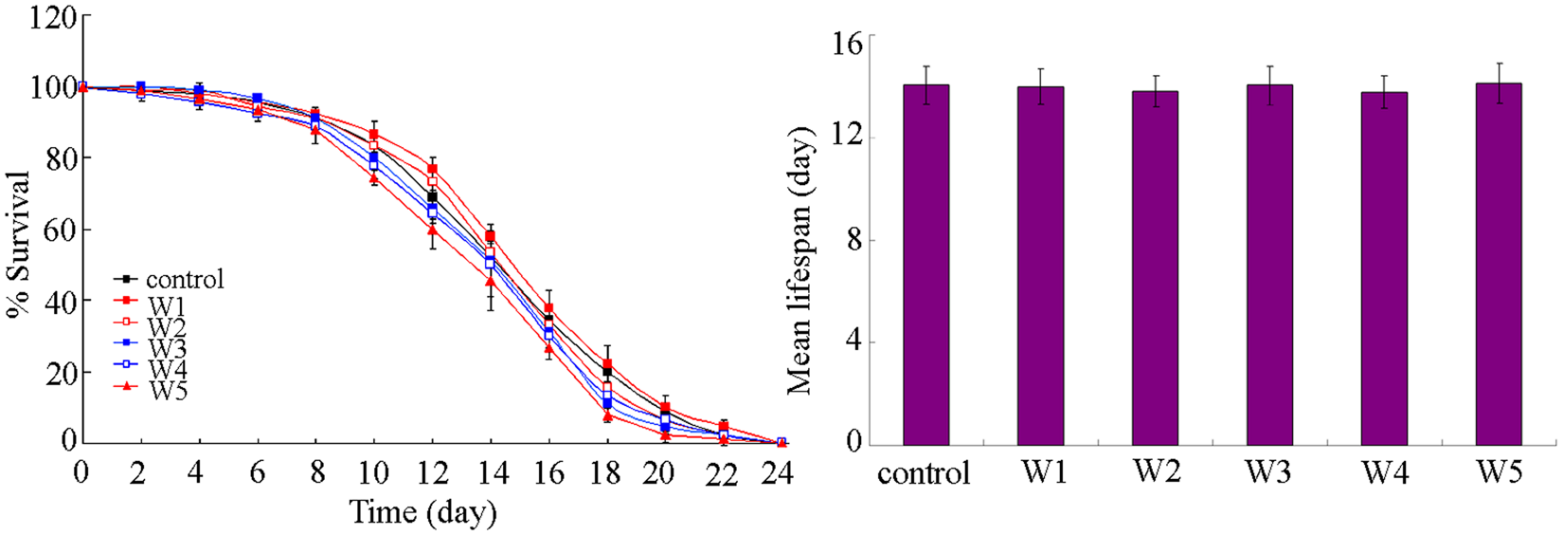
Effects of different surface water samples in the TGR region on lifespan of wild-type nematodes. Exposures were performed from L4-larvae for 24-h. Bars represent means ± SD.

We also selected the endpoint of lifespan to investigated the long-term effect of examine surface water samples in the TGR region on wild-type nematodes. After acute exposure, we found that all the examined five original surface water samples did not alter the lifespan (Fig. 6). Therefore, short-term exposure to the examined surface water samples in the TGR region may be not able to alter the lifespan.

### Toxicity assessment of surface water samples in *sod-2* or *sod-5* mutant nematodes

**Figure 7:**
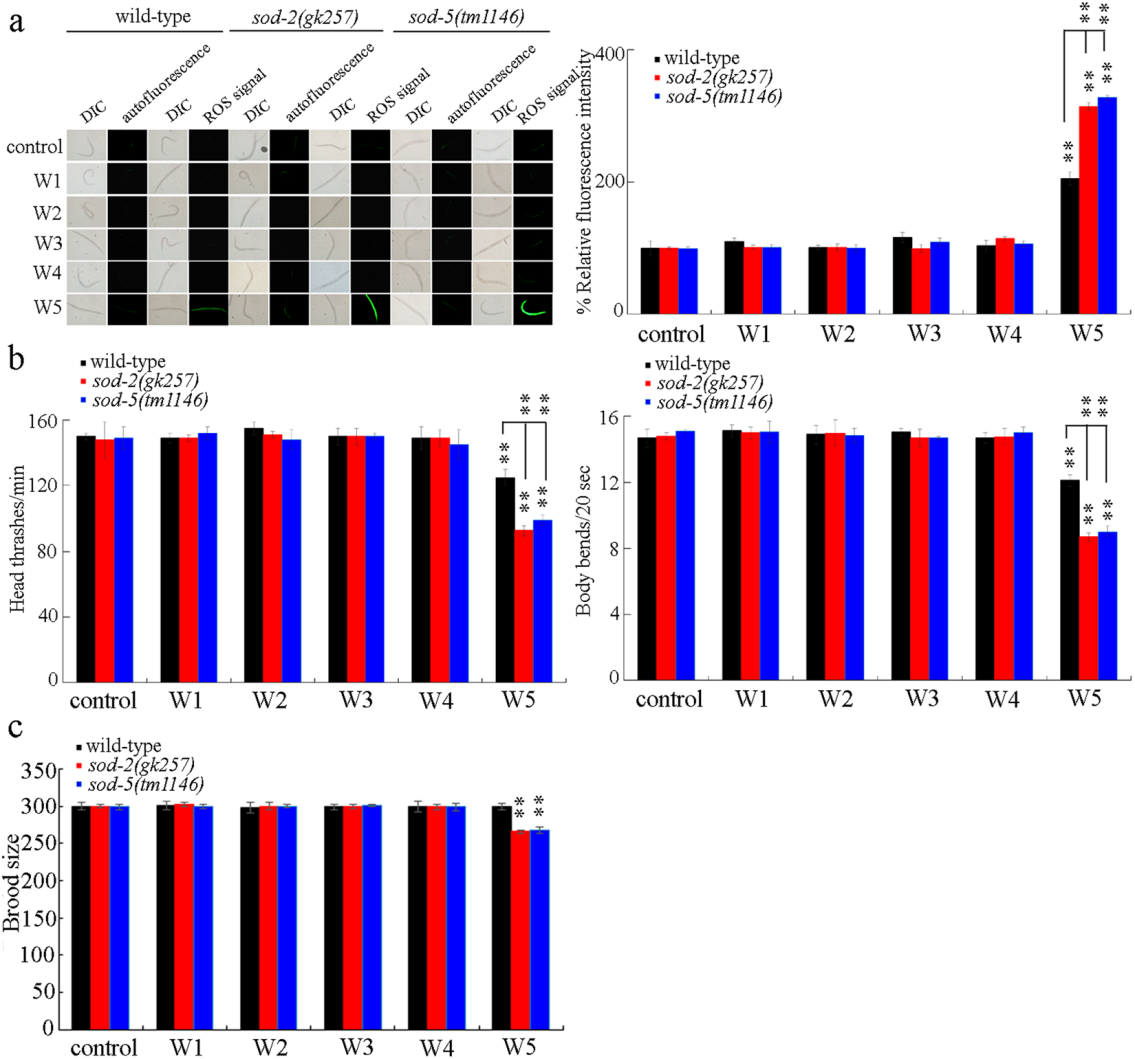
Effect of *sod-2* or *sod-5* mutation on the induction of ROS production (**a**), the locomotion behavior (**b**), and the brood size (**c**) in nematodes exposed to the examined different surface water samples in the TGR region. Exposures were performed from L4-larvae for 24-h. Bars represent means ± SD. ^**^P < 0.01 *vs* control (if not specially indicated).

Exposure to the surface water sample of W5 could induce the significant increase in *sod-2* and *sod-5* as indicated above. In *C*. *elegans*, mutation of *sod-2* or *sod-5* could cause the susceptibility to toxicity of environmental toxicants^60,61^. In *sod-2* or *sod-5* mutant nematodes exposed to the surface water sample of W1, W2, W3, or W4, we still did not detect their adverse effects in inducing intestinal ROS production, and on locomotion behavior or brood size (Fig. 7). In *sod-2* or *sod-5* mutant nematodes, all the examined five surface water samples still could not affect the survival, the body length, and the lifespan (data not shown). In contrast, the *sod-2* or *sod-5* mutant nematodes were susceptible to toxicity of surface water sample of W5 in the induction of ROS production and in the decrease of locomotion behavior (Fig. 7a and b). In *sod-2* or *sod-5* mutant nematodes exposed to the surface water sample of W5, we further found the significant reduction in brood size (Fig. 7c).

### Analysis on the chemical pollutants from the sample W5

**Figure 8:**
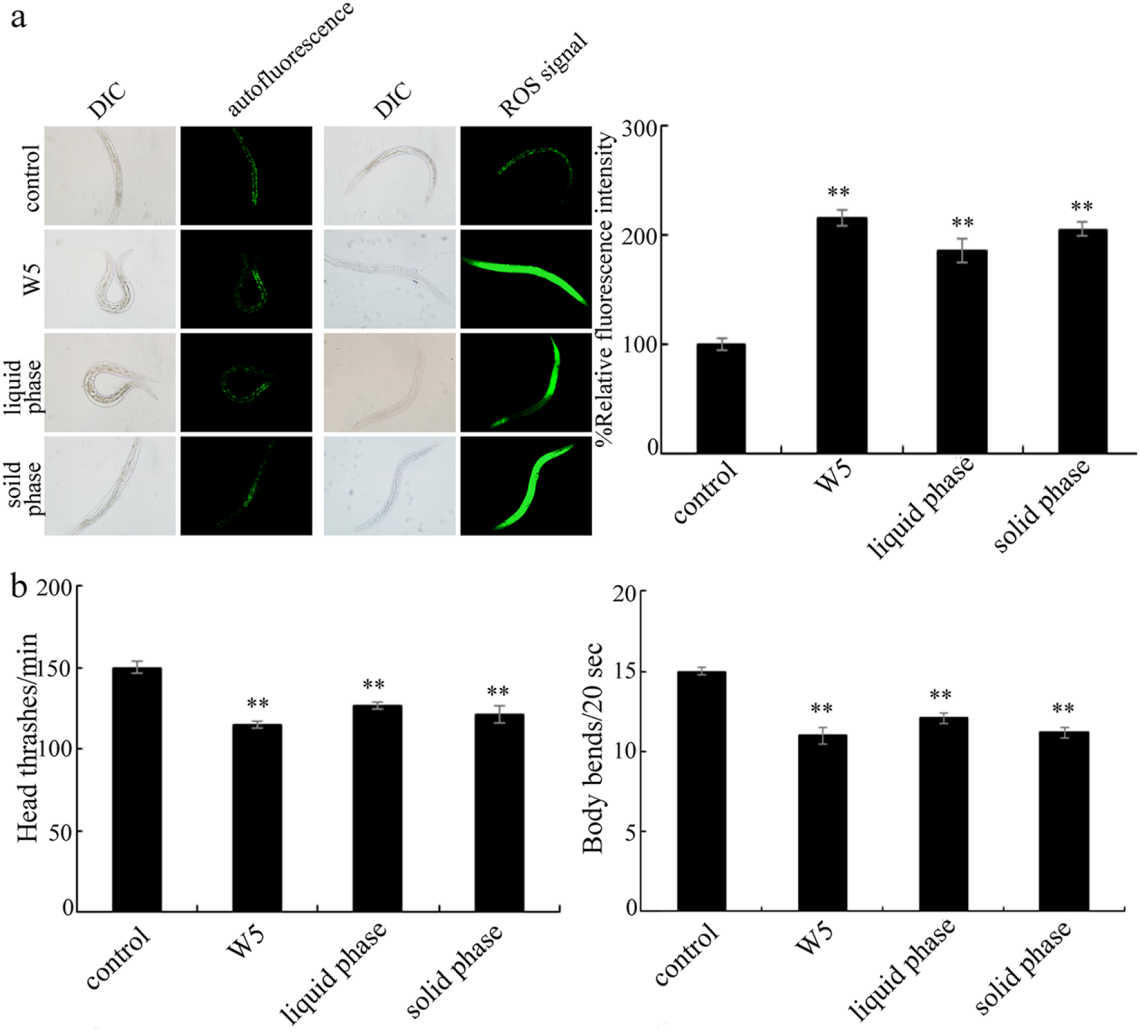
Effect of liquid phase or solid phase of the sample of W5 on the induction of intestinal ROS production (**a**) the locomotion behavior (**b**) in nematodes. Exposures were performed from L4-larvae for 24-h. Bars represent means ± SD. ^**^P < 0.01 *vs* control.

To determining the chemical pollutants from the sample of W5 in inducing toxicity on nematodes, we isolated the liquid phase and the solid phase from the sample of W5 by centrifugation at 10000 g for 10-min. After centrifugation, the liquid phase existed in the supernatant. The pellet was re-suspended with the equal volume of K medium to obtain the solution for the solid phase. After exposure, we found that both the liquid phase and the solid phase could cause the toxicity in inducing intestinal ROS production and on the locomotion behavior (Fig. 8).

In the liquid phase of sample of W5, the elemental analysis of surface water samples further showed Mg as the most abundant element, followed by K and Fe (Table S1). The examined surface water samples also contained moderate amounts of As, Hg, and Pb (Table S1). The content of Mg, K, As, Hg, or Pb in the surface water sample of W5 did not show the obvious difference from that in the other examined surface water samples (Table S1). We only found the moderate increase in the Fe content in the surface water sample of W5 compared with that in the other examined surface water samples (Table S1). However, acute exposure to Fe (0.242 mg/L) did not result in the obvious toxicity on nematodes using lethality, body length, intestinal ROS production, brood size, locomotion behavior, and lifespan as the endpoints (data not shown).

## Discussion

In this study, we performed the safety assessment on the effect of five original surface water samples from the TGR region using the model animal of *C*. *elegans*. The collected surface water samples are from upstream, shore water, downstream, and backwater area in Yangtze River in Wanzhou, Chongqing (Fig. 1). We selected the flood season (September) to collect the water samples in order to assess the possible effects of some chemicals released from the sediment or surrounding mountains. We performed the acute exposure in this study, because we noticed the existence of certain amount of bacterial community in the water in Yangtze River in TGR region^12,13^. Meanwhile, all the exposures were performed in the liquid solutions for nematodes at 20 °C.

Based on the toxicity assessment performed in this study, we found that short-term exposure to all the examined five original surface water samples from the TGR region did not significantly affect the survival, the body length, the brood size, and the lifespan wild-type nematodes (Figs 2, 5b and 6). These observations suggest that short-term exposure to all the examined original surface water samples from the TGR region may be not able to influence the survival, the development, the reproduction, and the longevity. Meanwhile, we also did not detect the obvious effect from acute exposure to surface water sample of W1, W2, W3, or W4 in inducing intestinal ROS production (Fig. 3a), in increasing GST-4::GFP expression (Fig. 3b), in enhancing intestinal permeability (Fig. 4), and in decreasing locomotion behavior as reflected by the endpoints of head thrash and body bend (Fig. 5a), which imply that the surface water samples collected from the upstream, shore water, or downstream may also be not able to influence the intestinal function, and the functions of motor neurons in nematodes after short-term exposure. Considering the existence of certain amount of bacterial community in the examined original surface water samples from the TGR region, so far it is difficult to determine the potential effects of long-term exposure to these examined original surface water samples on survival, development, reproduction, and longevity, as well as the effects on intestinal function and locomotion behavior.

We here detected the obvious intestinal ROS production (Fig. 3a), GST-4::GFP increase (Fig. 3b), enhanced intestinal permeability (Fig. 4), and decrease in locomotion behavior (Fig. 5a) in wild-type nematodes acutely exposed to the surface water sample of W5. The surface water sample of W5 was collected from the backwater area in Yangtze River in Wanzhou, Chongqing. Therefore, our results imply that short-term exposure to the surface water sample from the backwater area in Yangtze River in Wanzhou may potentially affect both the intestinal function as reflected by the enhanced intestinal permeability and the function of motor neurons as reflected by the decreased locomotion behavior in nematodes. One possibility is that the disrupted intestinal barrier may allow the entering of a large amount of pollutants from the sample of W5 into the targeted organs, such as the neurons.

Based on the analyzed physicochemical properties of collected five original surface water samples from the TGR region, we did not find the obvious difference in water level, longitude for sampling site, latitude for sampling site, altitude, water temperature, turbidity, pH value, total dissolved solids, biochemical oxygen demand, and chemical oxygen demand for the surface water sample of W5 from those for the surface water sample of W1, W2, W3, or W4 (Table S2). Therefore, the detected adverse effects of original surface water sample of W5 on intestinal function, locomotion behavior, and brood size may be not due to the difference in physicochemical properties from the examined other original surface water samples.

In the freshwater in the TGR region, dissolved heavy metals, and organic pollutants, such as endocrine-disrupting compounds, have been detected^7,9,11^. In the soil or the sediment in the TGR region, inorganic compounds, such as herbicide, and heavy metals, such as Hg and Cd, have also been detected^62–64^. Moreover, in the fresh water in the TGR region, two dominated bacterioplankton communities, Proteobacteria (39.26%) and Actinobacteria phyla (37.14%), were detected^13^. Recently, the evidence has been raised that the microbial structure and function can be affected by the environmental changes from the Three Gorges Dam^65,66^. In this study, we found that both the liquid phase and the solid phase contributed to the toxicity of the sample of W5 on nematodes (Fig. 8). Additionally, the observed acute toxicity on nematodes from the liquid phase of sample of W5 may not mainly due to the existence of heavy metals (Table S1), which implied that the potential pollutants in the liquid phase of sample of W5 may be the organic pollutants. The further identification of organic pollutants in the liquid phase and pollutants of bacterial community in the solid phase for the collected original surface water sample of W5 may be helpful for answering the reasons for the observed adverse effects of W5 on nematodes.

In this study, we further found that exposure to the surface water sample of W5 could significantly increase the expressions of *sod-2*, *sod-5*, and *clk-1*, and decrease the expression of *mev-1* (Fig. 3b). In *C*. *elegans*, *mev-1* and *clk-1* belong to the components constituting the molecular machinery in controlling the activation of oxidative stress^50,53^. This observation suggests that exposure to the surface water sample of W5 may result in intestinal ROS production by altering the molecular basis of oxidative stress. In *C*. *elegans*, increase in *sod-2* and *sod-5* may mediate a protection response for animals against the toxic effect from environmental toxicants or stresses^45,49^. Therefore, the surface water sample of W5 may also potentially activate a protection response in nematodes after exposure. Nevertheless, the activated protection response may be not able to counteract the adverse effect from the exposure to surface water sample of W5 in nematodes.

The *sod-2* or *sod-5* mutant was susceptible to the toxicity of environmental toxicants^60,61^. Moreover, we found that exposure to the surface water sample of W1, W2, W3, or W4 still could not cause the adverse effects in *sod-2* or *sod-5* mutant nematodes (Fig. 7). These results confirm the safe property for short-term exposure to the surface water sample of W1, W2, W3, or W4 on environmental organisms. Additionally, we observed that exposure to the surface water sample of W5 could result in the more severe toxicity in *sod-2* or *sod-5* mutant compared with wild-type nematodes (Fig. 7a and b), and mutation of *sod-2* or *sod-5* even caused the significant reduction in brood size (Fig. 7c). These observations demonstrate that the potential adverse effects of surface water sample of W5 can be further strengthened in environmental organisms with the susceptible property.

In conclusion, we employed the model animal of *C*. *elegans* to perform the safety assessment of the collected original surface water samples in the TGR region. Using lethal and some sublethal endpoints, we found that all the examined original surface water samples from the TGR region did not significantly affect the survival, development, reproduction, and longevity in wild-type nematodes. Exposure to the surface water sample of W1, W2, W3, or W4 also did not obviously induce the intestinal ROS production, alter the intestinal permeability, and affect the locomotion behavior. Additionally, exposure to the surface water sample of W1, W2, W3, or W4 could not cause the adverse effects on *sod-2* or *sod-5* mutant nematodes. In contrast, only exposure to the surface water sample of W5 could significantly induce the intestinal ROS production, enhance the intestinal permeability, and decrease the locomotion behavior. Moreover, mutation of *sod-2* or *sod-5* was susceptible to the adverse effects of the surface water sample of W5 on nematodes. The further analysis on the pollutants from the sample of W5 implies that both the liquid phase and the solid phase contributed to the observed toxicity in nematodes. Therefore, our results suggest that the surface water sample collected from the backwater area in Yangtze River in Wanzhou, Chongqing may have adverse effects on environmental organisms. Our data provide the important basis for the further systematic safety evaluation of the surface water samples collected in Yangtze River in the TGR region.

## Methods

### Study sites and sample collection

The surface water samples were collected from 5 sampling sites (W1, shore water; W2, upstream; W3, shore water; W4, downstream; and W5, backwater area) in Wanzhou, Chongqing. The sampling season was selected in the flood season (September 18, 2017). At this season, the bacterioplankton community was generally higher than that in the impoundment season^13^. Samples were stored in a car refrigerator with about 0 °C after collection. Water samples were collected according to the standard method^67^, and analyzed after being transported back to the laboratory in a cooler.

Water temperature and pH were measured *in situ* using a portable SG2-ELK pH meter (Mettler-Toledo Co., Shanghai, China). Total dissolved solids were measured *in situ* using a portable SG3 conductivity meter (Mettler-Toledo Co., Shanghai, China). Turbidity was measured using a 1900C turbidimeter (Hach Co., Colorado, USA). Biochemical oxygen demand was analyzed according to the Chinese standard examination method for water quality (HJ 505–2009). Chemical oxygen demand was analyzed according to Chinese standard examination method for water quality (GB 11914–89). The daily flow and water level data of sampling sites were obtained from Yangtze River Hydrological Network (http://www.cjh.com.cn). The related information for the collected surface water samples in TGR region is shown in Table S2. Major elements of surface water samples were determined by inductively coupled plasma atomic emission spectroscopy (ICP, GE Co., USA).

### C. elegans maintenance

The used nematode strain was wild-type N2, mutants of *sod-2(gk257)* and *sod-5(tm1146)*, and transgenic strains of *zIs356*[P*daf-16::daf-16a/b::GFP*], and CL2166/*dvIs19*[gst-4::GFP]. Gravid nematodes were transferred into centrifuge tubes, and lysed with bleaching solution to separate the eggs and the animals. Age synchronous population of L4-larvae was obtained as described^68^.

### Exposure

Exposures were performed from L4-larvae for 24-h (acute exposure) in liquid solutions in the presence of food (OP50) at 20 °C. After exposure, nematodes were used for toxicity assessment with lethality, body length, intestinal ROS production, brood size, locomotion behavior, and lifespan as the endpoints. Control exposure was the treatment with liquid K medium. The exposure was performed in triple replicates.

### Toxicity assessment

For the lethality assay, the nematodes are judged to be dead if they can not respond to the stimulus using a small metal wire. Fifty nematodes were examined per treatment.

Body length was used to assess the growth of nematodes. Body length was determined by measuring the flat surface length of nematodes using Image-Pro^®^ Express software. Thirty nematodes were examined per treatment.

Intestinal ROS production was used to assess the oxidative stress in intestine^69^. The method was performed as described previously^70^. The 5′,6′-chloromethyl-2′,7′dichlorodihydro-fluorescein diacetate (CM-H_2_DCFDA) can detect the presence of intracellular produced ROS species. The examined nematodes were incubated with 1 μM CM-H_2_DCFDA solution for 3 h in the dark. The examined nematodes were analyzed at 488 nm of excitation wavelength and at 510 nm of emission filter under a laser scanning confocal microscope. Relative fluorescence intensity of ROS signals was semi-quantified in comparison to the intestinal autofluorescence. Thirty nematodes were examined per treatment.

The method for analysis for head thrash and body bend was performed as described previously^71,72^. The behaviors were analyzed under the dissecting microscope by eyes. A head thrash is defined as a change in the direction of bending at the mid body, and a body bend is defined as a change in the direction of the part of the nematodes corresponding to the posterior bulb of the pharynx along the y axis, assuming that nematode was traveling along the x axis. Fifty nematodes were examined per treatment.

The method for brood size was performed as described previously^73,74^. The number of offspring at all stages beyond the egg was counted. Thirty nematodes were examined per treatment.

Lifespan was used to reflect the long-term effect of certain toxicants in nematodes. The method was performed as described previously^75^. After the exposure, we started to record the lifespan of nematodes. During the lifespan recording, the hermaphrodites were transferred daily for the first 7 days of adulthood in order to avoid the possible effect from the progeny. Nematodes were checked every day. Fifty nematodes were examined per treatment. Graphs are representative of at least three trials.

### Reverse-transcription and quantitative real-time polymerase chain reaction (qRT-PCR)

After synthesis of the cDNA, relative transcriptional expressions of certain genes were analyzed in an ABI 7500 real-time PCR system (Biotium, USA). Relative quantification of targeted genes in comparison to a reference gene of *tba-1* encoding a tubulin was determined. Primer information is provided in Table S3.

### Intestinal permeability assay

The examined nematodes were suspended in blue food dye (5.0% wt/vol in water) in the presence of OP50 for 3 h. After that, the nematodes were transferred onto normal NGM plates seeded with OP50 to analyze the blue food dye in the body cavity using a microscope. Twenty nematodes were examined per treatment.

### Statistical analysis

Statistical analysis was performed using SPSS 12.0 software. The differences between groups was determined using analysis of variance (ANOVA). Probability level of 0.05 was considered as the statistically significant. The lifespan data were analyzed using a 2-tailed 2 sample *t*-test (Minitab Ltd, Coventry, UK).

## Electronic supplementary material

Supporting Information
