# Author Correction: Toxicity evaluation of Wanzhou watershed of Yangtze Three Gorges Reservoir in the flood season in *Caenorhabditis elegans*

**DOI:** 10.1038/s41598-018-34064-9

**Published:** 2018-10-18

**Authors:** Guosheng Xiao, Li Zhao, Qian Huang, Junnian Yang, Huihui Du, Dongqin Guo, Mingxing Xia, Guangman Li, Zongxiang Chen, Dayong Wang

**Affiliations:** 1 College of Biology and Food Engineering, Chongqing Three Gorges University, Wanzhou, 404100 China; 2 Medical School, Southeast University, Nanjing, 210009 China; 3 Wanzhou Entry-Exit Inspection and Quarantine Bureau, Wanzhou, 404100 China

Correction to: *Scientific Reports* 10.1038/s41598-018-25048-w, published online 30 April 2018

The original version of this Article contained an error in the title of the paper, where the word “Reservoir” was incorrectly given as “Reservior”. This has now been corrected in the PDF and HTML versions of the Article, and in the accompanying Supporting Information file.

