# Supplementary material for: Toxicity evaluation of Wanzhou watershed of Yangtze Three Gorges Reservoir in the flood season in Caenorhabditis elegans
Source: Sci Rep. 2018 Apr 30;8:6734. doi: 10.1038/s41598-018-25048-w (PMC5928115; doi:10.1038/s41598-018-25048-w)
Supplement: Supplementary file 1 — Supporting Information [file 41598_2018_25048_MOESM1_ESM.pdf]

Toxicity evaluation of Wanzhou watershed of Yangtze Three Gorges Reservoir in the flood season in *Caenorhabditis elegans*

Guosheng Xiao[1, *], Li Zhao[2, *], Qian Huang[1], Junnian Yang[1], Huihui Du[1], Dongqin Guo[1], Mingxing Xia[3], Guangman Li[3], Zongxiang Chen[3] & Dayong Wang[2, **]

[1]College of Biology and Food Engineering, Chongqing Three Gorges University, Wanzhou 404100, China

[2]Medical School, Southeast University, Nanjing 210009, China

[3]Wanzhou Entry-Exit Inspection and Quarantine Bureau, Wanzhou 404100, China

[*]They contributed equally to this work.

[**]Correspondence and requests for materials should be addressed to D.W. (email: dayongw@seu.edu.cn).

Supporting Information:

Table S1. Major elemental analysis in the collected surface water samples in TGR region

	W1	W2	W3	W4	W5
Cd (μg/L)	not detectable	not detectable	not detectable	not detectable	not detectable
Pb (μg/L)	0.5637	0.2914	0.0527	0.187	0.1181
Hg (μg/L)	0.014	0.023	0.022	0.016	0.019
As (μg/L)	0.973	0.967	0.994	1.009	1.003
Zn (μg/L)	not detectable	not detectable	not detectable	not detectable	not detectable
Cu (μg/L)	not detectable	not detectable	not detectable	not detectable	not detectable
Mn (μg/L)	not detectable	not detectable	not detectable	not detectable	not detectable
Cr (μg/L)	not detectable	not detectable	not detectable	not detectable	not detectable
Fe (mg/L)	0.172	0.156	0.178	0.174	0.242
K (mg/L)	2.674	2.678	2.61	2.773	2.902
Mg (mg/L)	7.43	7.431	7.479	7.392	7.427

Table S2. Information for the collected surface water samples in TGR region

	W1	W2	W3	W4	W5
Water level (m)	163.5	163.5	163.5	163.5	163.5
Longitude for sampling site	108°23'36"	108°25'15"	108°25'15"	108°24'2"	108°23'25"
Latitude for sampling site	30°47'30"	30°45'39"	30°48'30"	30°49'22"	30°47'45"
Altitude (m)	160	170	150	150	150
Water temperature (°C)	27.3	28	28	26	26.7
Turbidity (NTU)	53.5	44.2	44.4	46.9	47.9
pH value	8.106	8.124	8.081	8.024	8.007
Total dissolved solids (ppm)	153	149	142	148	156
Biochemical oxygen demand (mg/L)	7.56	7.34	7.78	7.65	7.26
Chemical oxygen demand (mg/L)	12.4	11.2	13.3	12.7	11.6

Table S3. Primers used for quantitative real-time polymerase chain reaction

Gene	Forward primer	Reverse primer
tba-1	TCAACACTGCCATCGCCGCC	TCCAAGCGAGACCAGGCTTCAG
clk-1	CACATACTGCTGCTTCTCGT	TGAACCAACAGATGAACCTT
gas-1	CTTGGTCTTTGGCTGTTGA	CTTGGTCTTTGGCTGTTGA
isp-1	GCAGAAAGATGAATGGTCC	CAGAAGCGTCGTAGTGAGA
mev-1	GGAATTCGCTTCTTAGGAT	GCAGTCTTGTTGCTCTTGT
sod-1	ACGCTCGTCACGCTTTAC	TCTTCTGCCTTGTCTCCG
sod-2	GGCATCAACTGTCGCTGT	ACAAGTCCAGTTGTTGCC
sod-3	TGACATCACTATTGCGGT	GGGACCATTCCTTCCAAA
sod-4	CACCAGATGACTCGAACA	AATGAGGCAAGAGAGTCG
sod-5	AAAGTAGAGTCGAAACGTGCTG	TGAAGTCCTGGTGACAATCCCT
daf-16	ACATTGCTCGAAGTGCCGAA	CATTGCTGTCGACCCGTTTG
pmk-1	CGAAGAACGAGCAGAAGAGT	TCCAGTTCAATATGGTGGTG
egl-8	GCAGAGGGATAGCATTCA	TCTTGTGGCACTGGACTT
eps-8	CTGTTCTCCGTCGCCTTGAT	GGTGGAGTACGGAATGGTGG
act-5	CAGGGAGTGATGGTCGGTAT	CGGTAAGGAGAACTGGGTGT
opt-2	CGCGTCACTTGCCTTTTTGA	TTTGACGGCACATGGCAAAG
nhx-2	CTCAAACCATCACTGGGACA	CACCACGAAGACCTCCATAA
pgp-1	TGCGAAAGCCGGAAAAGTTG	TTTGAAGCCTGCATTGCACC
let-413	GGACCTCCCTGACACGAT	CACCAAGAAATGCTCCTC
pkc-3	GTTTGAAATGATGGCTGGTC	GCTGGATGATATGGAGGTGC
erm-1	CTTCTACGCTCCACGACTCC	TTCCTCCACGGATTTGACGG
pgp-3	TGGGACTTCCTGACGGTTAC	CGTTTGATGGGTTCCTTCTT
